# Non-neoplastic astrocytes: key players for brain tumor progression

**DOI:** 10.3389/fncel.2023.1352130

**Published:** 2024-01-16

**Authors:** Myriam Catalano, Cristina Limatola, Flavia Trettel

**Affiliations:** ^1^Laboratory of Neuroimmunology, Department of Physiology and Pharmacology, Sapienza University of Rome, Rome, Italy; ^2^IRCCS Neuromed, Pozzilli, Italy

**Keywords:** non-neoplastic astrocytes, primary brain tumors, metastatic brain tumors, glioma, tumor microenvironment

## Abstract

Astrocytes are highly plastic cells whose activity is essential to maintain the cerebral homeostasis, regulating synaptogenesis and synaptic transmission, vascular and metabolic functions, ions, neuro- and gliotransmitters concentrations. In pathological conditions, astrocytes may undergo transient or long-lasting molecular and functional changes that contribute to disease resolution or exacerbation. In recent years, many studies demonstrated that non-neoplastic astrocytes are key cells of the tumor microenvironment that contribute to the pathogenesis of glioblastoma, the most common primary malignant brain tumor and of secondary metastatic brain tumors. This Mini Review covers the recent development of research on non-neoplastic astrocytes as tumor-modulators. Their double-edged capability to promote cancer progression or to represent potential tools to counteract brain tumors will be discussed.

## 1 Introduction

Although neurons are the excitable and firing cells of the brain driving the nervous system signaling, glial cells in the brain parenchyma are key players for the correct functioning and the homeostasis of the central nervous system. Among glial cells, “star-like” astrocytes are the cells whose relative number, size, number of ramified processes and volume increased with phylogeny and brain complexity (Nedergaard et al., 2003). Depending on the different regions, astrocytes represent 20−40% of all brain cells (Herculano-Houzel, 2014) and show different morphology, ranging from protoplasmic to spherical shape (Emsley and Macklis, 2006; Oberheim et al., 2006). In the brain, each astrocyte occupies a specific territory, with less of 5% of overlap with neighboring astrocytes (Ogata and Kosaka, 2002). Within their specific competence territory, astrocytes contact blood vessels and up to hundred-thousands of different synapses (Halassa et al., 2007); moreover, due to the presence of connexin gap junctions between different astrocytes, these cells are organized in networks (D’Ambrosio et al., 1998; Giaume et al., 2010) that appear to be organized in functional domain (Giaume et al., 2010). Generally believed to mainly have a supportive function (Kettenmann and Ransom, 2005), astrocytic cells play many active roles. During development, astrocytes play a role in guiding the migration of neuronal axons and neuroblast (Powell and Geller, 1999), and the formation of developing synapses (Ullian et al., 2001; Christopherson et al., 2005); moreover, they can drive microglial synapse engulfment, or actively engulf synapses and sculpt neuronal circuits (Chung et al., 2013; Vainchtein et al., 2018). With their terminal processes (end-feet), astrocytes contribute to the formation and maintenance of brain-blood integrity (Abbott, 2002); thank to the presence of several plasma membrane transporters, during neuronal activity they can buffer extracellular K^+^ concentration and water content (Simard and Nedergaard, 2004), regulate the extracellular pH and remove excessive glutamate from the synapses (Rose et al., 2018). Astrocytes sense neuronal activity via metabotropic neurotransmitter receptors, and are able to provide energy substrate to neurons through the so call “astrocyte-neuron lactate shuttle” (Magistretti and Pellerin, 1999); in addition, astrocytic networks can support the high energy demand of neuronal activity, also at site distant from blood vessels (Rouach et al., 2008), thus ensuring glia-neurons metabolic coupling necessary for memory formation (Suzuki et al., 2011; Gao et al., 2016). Also, astroglial endfeets that enwrap blood vessels are characterized by high levels of connexins expression (Rouach et al., 2008) and Ca^2+^ signaling within astrocytes can trigger the release of vasoactive molecules that modulate local or regional cerebral blood flow (Koehler et al., 2009; Institoris et al., 2022). Being part of the “tripartite” synapse (Araque et al., 1999), astrocytes respond to neurotransmitter release by presynaptic terminals with an increase in intracellular Ca^2+^, and consequent release of “gliotransmitters” that can act regulating synaptic plasticity at local synapse (Fellin et al., 2006; Di Castro et al., 2011). Moreover, intracellular calcium increase can be spread to other connected astrocytes (Bazargani and Attwell, 2016; Goenaga et al., 2023) resulting in neurotransmitter release and modulation of synapses at the level of network activity (Fellin, 2009; Miguel-Quesada et al., 2023).

In non-physiological conditions, such as CNS (central nervous system) injuries, disease or brain tumor, astrocytes lose their “quiescent” state, become “reactive” and undergo changes in molecular expression, progressive cellular hypertrophy and in some cases also proliferation and scar formation (Lukaszevicz et al., 2002; Sofroniew, 2009; Faideau et al., 2010; Acevedo-Arozena et al., 2011; Cuevas-Diaz Duran et al., 2019; Makarava et al., 2019). These changes are regulated in a context-specific manner, and lead to altered astrocytic activities, either loss or gain of functions, that can be either detrimental or beneficial to the brain (Sofroniew, 2005).

## 2 CNS primary tumors originating from astrocytes

CNS primary tumors are the most frequent in children between 0 and 14 years of age and are the eighth most frequent in adults (van den Bent et al., 2023). These tumors are extremely heterogeneous, depending not only on the tissue of origin but also on the genetic and/or molecular modifications that characterize them and on the ethnicity of the affected population; all these aspects define the average outcome of patients (Louis et al., 2021). A first distinction is between malignant and non-malignant tumors; the first ones are able to invade the surrounding tissue and have a terrible outcome; the others are classified based on histological and molecular characteristics. Among the malignant CNS tumors, gliomas are the most common, and glioblastoma (GBM) is the most aggressive and frequent primary malignant CNS tumor, with the prognosis of an overall survival of 7−17 months after surgical removal (Molinaro et al., 2020). Among the non-malignant tumors, the most common is meningioma (Louis et al., 2016).

Despite malignant brain tumors can originate from neuronal stem cells or oligodendrocyte precursor cells, astrocytes represent the cellular origin at least for a defined number of cases (Zong et al., 2015). In fact, a specific mouse model carrying co-deletion of the tumor suppressor Tp53, Pten and Rb1 genes was created using the site-specific recombinase technology (Cre-Lox/GFAP) in adult mice (Chow et al., 2011). This model shows deletion of the three tumor suppressor genes widespread in mature astrocytes but also in a subpopulation of GFAP-expressing neuronal stem cells (NSCs) in the brain proliferative niches (subventricular zone and subgranular layer). Most tumors grew in these niches but more than 20% of tumors appeared in the non-proliferative areas (specifically in cortex, brainstem, cerebellum, and spinal cord). These data demonstrate that mature astrocytes are a cell type from which malignant CNS tumors arise even if the majority originates from stem cells.

Astrocytes can originate malignant CNS tumors not only by alteration of their proliferation and/or cell survival process, but also in case of alterations in the differentiation state maintenance. Indeed, it has been shown that astrocytes dedifferentiated into NSCs - after stimulation with TNFα - become susceptible to the process of cancerization by irradiation; in contrast mature astrocytes do not undergo transformation upon the same oncogenic stress (Dufour et al., 2009).

## 3 Metastatic brain tumors

Metastatic brain tumors are secondary tumors that develop from cells of a primary systemic tumor that invade the brain and are often the main cause of mortality (Achrol et al., 2019). The most common primary tumors that develop brain metastasis are the lung (40−50%), breast (15−20%), skin (5−10%), and gastrointestinal (4−6%) tumors, and most patients develop more than one brain lesion. Similar to glioma, standard of care to treat brain metastasis is the surgical removal, followed by radiation- and chemo- therapies; nevertheless, these treatments have a reduced efficacy, with most patients developing local recurrence in less than one year (Brastianos et al., 2013). Brain metastasis formation requires several steps such as detachment from the primary tumor, invasion of surrounding tissue, intravasation in blood vessel, dissemination and arrest in brain capillary, extravasation through non-fenestrated capillaries, colonization of surrounding tissue and local proliferation and neo-angiogenesis (Svokos et al., 2014). The interaction between circulating tumor cells and blood–brain barrier (BBB) components is mainly mediated by cytokines and chemokines (Seike et al., 2011); tumor cell proliferation and angiogenesis in the brain depend on the release of local growth factors (Hoshide and Jandial, 2017).

## 4 Brain tumor/non-neoplastic astrocyte crosstalk

It is well established that the crosstalk between brain tumor cells and the surrounding microenvironment is determinant for tumor progression. In particular 50% of glioma tumor mass is made by the infiltration of brain-resident microglia, and peripheral macrophages (Hambardzumyan et al., 2016) that actively contribute to tumor proliferation and invasion, but also to the formation of an immune suppressive environment (Catalano et al., 2020). Indeed, other infiltrating immune cells are present, these are primarily T lymphocytes, and also rare NK, dendritic and B cells (Gieryng et al., 2017). In particular the infiltrating T cells display an exhausted phenotype and undergo programmed cell death (Woroniecka et al., 2018), resulting in the inability to contrast the tumor.

In this context, astrocytes in the proximity of the tumor became reactive (Nagashima et al., 2002; O’Brien et al., 2013) as a consequence of their ability to sense the tumor and to engage a crosstalk with the tumor microenvironment (TME), becoming part of it. Reactive astrocytes appear to play an important role in supporting glioma growth since, as it has been recently found, genetic ablation of tumor-associated astrocytes in glioma GL261 bearing mice, not only stalls GBM progression but drives the tumors into regression and prolongs animal survival (Perelroizen et al., 2022). The ability of astrocytes to modulate tumor growth seems to depend on the phenotype of glioma cells. In fact, if the glioma is minimally invasive (i.e., U87MG glioma cell line), astrocytes are able to totally block its migration and probably to stimulate a robust immune response. If the glioma is highly invasive (i.e., LN229 glioma cell line), astrocytes increase the migratory capacity of tumor cells by inducing over-expression of genes related to migratory signaling pathways, such as STAT3 (signal transducer and activator of transcription) and HGF/MET (hepatocyte growth factor/mesenchymal-epithelial transition factor) (Cui et al., 2023).

Also in the case of brain metastasis derived from a primary systemic tumor, the interaction between metastatic cells and the surrounding brain TME plays a fundamental role in the establishment of brain metastasis (Gwak, 2023). Among cells of the TME, reactive astrocytes are the most active host cell population, that immediately localizes to individual invading tumor cells and continuously associates with growing metastatic lesions (Lorger and Felding-Habermann, 2010). Moreover, in a model of spontaneous brain metastasis, in immunocompetent mice, Schwartz et al. (2016) demonstrated that astrocytes acquire a proinflammatory phenotype in the brain metastatic niche before the formation of macrometastasis.

**FIGURE 1:**
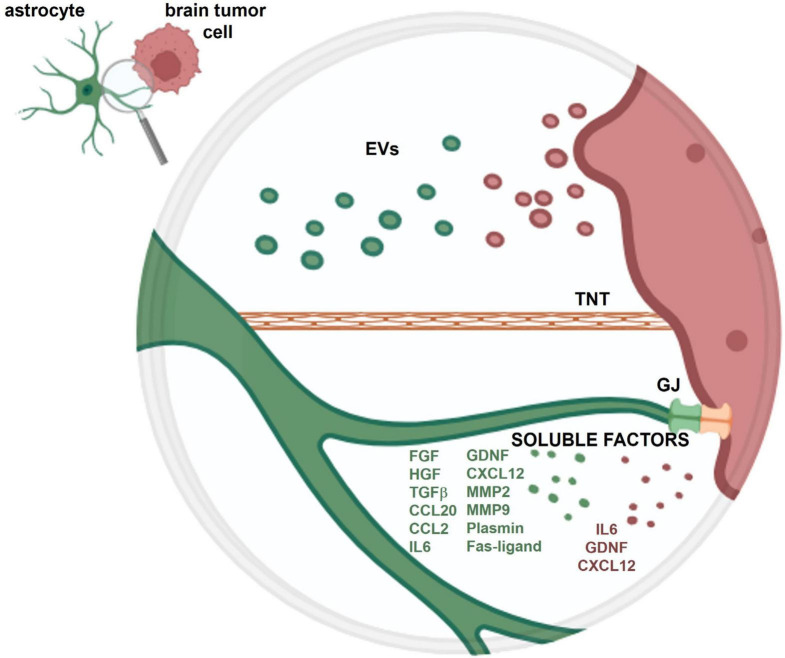
Schematic representation of the crosstalk between an astrocyte and a brain tumor cell. The crosstalk is mediated by soluble factors and extracellular vesicles (EVs) released from both cell types and through the direct contact between cells, via gap junctions (GJ) and tunneling nanotubes (TNT). More detailed information on the role of these different types of crosstalk is provided in the present review.

The crosstalk between astrocytes and tumoral cells within the brain is mediated by the release of soluble factors, the release of extracellular vesicles (EVs) and through the direct contact between cells, due to gap junctions or to tunneling nanotubes (Figure 1).

### 4.1 Crosstalk *via* secreted molecules

The secretome of astrocytes depends on the surrounding milieu. For this reason, the mechanisms underlying the interaction between astrocytes and glioma cells are not linear. In fact, the release of many molecules by astrocytes is affected by the ongoing TME and by factors released by tumor cells, and largely contributes to the growth and invasion of the glioma and metastatic brain tumors.

Astrocytes stimulated with the ligand of the receptor activator of nuclear factor kappa-B (RANKL), that is overexpressed by invasive glioma cell lines (Jin et al., 2011), release factors that induce glioma cells invasion such as fibroblast growth factor (FGF), hepatocyte growth factor (HGF), and transforming growth factor β (TGF-β) (Kim et al., 2014). Under hypoxic conditions (that resemble the hypoxic microenvironment in which astrocytes and glioblastoma coexist), astrocytes increase the release of the chemokine CCL20. Astrocytic CCL20 promotes glioma cell invasion through the increase in tumor cell expression of the hypoxia-inducible factor HIF-1α (Jin et al., 2018). Similarly, the chemokine CCL2, that in the brain is mainly produced by astrocytes, turned out to be a key element for metastatic brain tumor cell migration both *in vitro* and *in vivo* (Hajal et al., 2021). Cytokine IL6 represents another example of the reciprocal activation between glioma and astrocytes; it is released by the tumor and acts in a paracrine way increasing its own secretion by astrocytes (Liu et al., 2010, Chen W. et al., 2016). Increased release of IL6 promotes migration and invasion of glioma cells through the activation of the transcription factor STAT3 (Chen et al., 2020). This reciprocal activation has not been highlighted for GDNF (glial-derived neurotrophic factor) that does not exert paracrine effect to induce astrogliosis (Ku et al., 2013), but when released by astrocytes enhances the growth and invasion of the tumor (Shabtay-Orbach et al., 2015), and when released by the tumor acts autocrinally to strengthen glioma growth (Lu et al., 2010).

In the central nervous system astrocytes are among the major producers of CXCL12 (Trettel et al., 2020) and express its specific receptor CXCR4. CXCL12 with an autocrine effect activates CXCR4 promoting astrocyte proliferation through the ERK1-2/PI-3K (extracellular signal-regulated kinase 1-2/phosphatidylinositol 3-kinase) signaling pathway (Bajetto et al., 1999; Barbero et al., 2002). Glioma cells also release CXCL12 and overexpress its receptor CXCR4 (Salmaggi et al., 2005; Bian et al., 2007). Stimulation of this axis activates not only ERKs but also AKTs proliferative pathways promoting tumor growth and invasion (Barbero et al., 2003; Rubin et al., 2003). CXCL12 is also able to attract CXCR4 + myeloid derived suppressor cells to create an immunosuppressive microenvironment that favors the growth of glioma (Alghamri et al., 2022), as reported for other tumors (Obermajer et al., 2011; Benedicto et al., 2018). CXCL12 has higher affinity for the receptor CXCR7 (Esencay et al., 2013) whose high expression correlates with poor glioblastoma patient survival (Deng et al., 2017) and similarly to CXCR4 activation, triggers glioma proliferation and invasion (Liu et al., 2015). Astrocytes express CXCR7 (Odemis et al., 2010), whose activation reinforces the CXCR4-mediated proliferative signaling. The expression of CXCR7, and not of CXCR4, on astrocytes is modulated by microenvironmental conditions, such as hypoxic conditions induced by tumoral cells. This distinction highlights the complex role of the trio CXCL12/CXCR4/CXCR7 in the bidirectional interaction between astrocytes and glioma cells. Of note, there is another aspect that generates greater complexity in this interaction. Astrocytes under specific stimuli are able to produce the matrix metalloproteinases MMP2 and MMP9 (Ogier et al., 2006), both overexpressed in GBM patients and correlating with patient poor prognosis. These enzymes also mediate the proteolytic processing of CXCL12 into the specific CXCL12(5-67) peptide, a neurotoxic protein that binds CXCR3, whose expression in GBM patients also correlates with a patient’s poor prognosis. Even if the direct effect of the chemokine peptide CXCL12(5-67) on glioma cells has not yet been evaluated, CXCR3 activation increases glioma cell invasion whilst CXCR3 downregulation inhibits glioma stem cells viability (Pu et al., 2015; Boyé et al., 2017).

Astrocytes are among the brain parenchymal cells that first make contact with extravasated metastatic cells. Reactive astrocytes close to extravasating metastatic tumor cells in the brain, also overexpress and release MMP9 favoring the development of brain metastasis (Lorger and Felding-Habermann, 2010). Using different types of breast cancer or lung cancer cell lines, to induce brain metastatization in mice, it has been shown that, by sensing brain infiltrating cancer cells, astrocytes became reactive and attempt to defend against metastatic invasion by releasing both Plasmin (PA) and Fas-ligand, that induce cancer cell death. Moreover plasmin induces the destroying of L1CAM (L1 cell adhesion molecule) expressed by cancer cells, preventing their ability to coopt brain capillaries. (Valiente et al., 2014). However, some metastatic cells can express high levels of antiPA-serpin (that prevents PA formation) preventing cell death and fostering vascular cooption (Valiente et al., 2014).

In addition, it has been shown that proinflammatory astrocytes are instigated to overcome brain tissue damage due to the entrance of metastatic cells into the brain. Later on, reactive astrocytes are hijacked by brain-metastasizing tumor cells in order to express *SerpinE1* and *SerpinA3N* genes, that support metastasis growth (Schwartz et al., 2016).

### 4.2 Crosstalk *via* extracellular vesicles

In addition to the secretome, that acts close to the cell of origin, EVs that can act also far from the donor cell. These particles are made up of a phospholipid bilayer that contains protein, lipid and genetic materials which is completely transferred to the recipient cell. Tumors and among them gliomas, release huge amounts of EVs as a tumorigenic mechanism, being their content able to activate transforming signaling pathways in target cells; for example, tumor-derived EVs induce transformed features to normal adjacent cells (i.e., fibroblasts, stromal and epithelial cells) such as anchorage-independent growth and enhanced or aberrant growth capability (Webber et al., 2010; Antonyak et al., 2011; Paggetti et al., 2015). Glioma-derived EVs, as described in general for cancer-derived vesicles, could also be shared between tumoral cells. Tumor-derived EVs transfer drug-resistance molecules from drug-resistant cells to drug-sensitive ones as observed in breast cancer (Lv et al., 2014).

In addition, they activate macrophages, B and NK cells, induce maturation of dendritic cells and promote generation of myeloid-derived suppressor cells (Liu et al., 2006; Valenti et al., 2006; Yu et al., 2007; Clayton et al., 2008). All these effects belong to the pleiotropic mechanism aimed by tumor EVs to promote an immune-suppressive microenvironment supporting the cancer development. Glioma-derived EVs target different immune cells supporting their defective response, one of the major hallmarks of tumor occurrence. They transfer onco-miRs as for example miR155, miR214, miR21 (Zonari et al., 2013; Feng and Tsao, 2016; Abels et al., 2019; Yang et al., 2019; Xu et al., 2021; Orso et al., 2023) or tumorigenic transcription factors (such as *Stat3*) into tissue-resident microglia, infiltrating myeloid-derived macrophages (Gabrusiewicz et al., 2018; Johnson et al., 2018; Xu et al., 2021) and tumor-infiltrating regulatory T cells (Li et al., 2017).

With respect to astrocytes, GBM EVs stimulate astrocyte release of a huge amount of growth factors, cytokines and chemokines (few examples are TNFα, CCL20, IL10 and CCL2, see above) (Oushy et al., 2018), that mediate autocrine effect promoting astrocytes migration and paracrine effects inducing tumor cells migration and invasion (Kucharzewska et al., 2013; Mu et al., 2013; Taheri et al., 2018). Tumor EVs induce an upregulation of genes important for extracellular matrix remodeling (i.e., MMP2 and MMP9). GBM EVs also show transforming capability toward astrocytes, perhaps by transferring oncogenes how demonstrated for the oncogenic form of the epidermal growth factor receptor (EGF), called EGFRvIII, horizontally transferred among glioma cells to induce the activation of EGFRvIII-dependent oncogenes (Al-Nedawi et al., 2008).

The effect of EVs released by astrocytes in most cases enhances brain tumor development. In fact, it has been demonstrated that astrocytes derived EVs transfer miRs that inhibit the important tumor suppressor PTEN in metastatic tumor cells (Zhang et al., 2015). Notably, PTEN loss is responsible for the increased release of CCL2 (Hajal et al., 2021) that autocrinally reinforces the migration of tumor cells. Astrocytes derived EVs also contain factors such as fibroblast growth factor-2 and vascular endothelial growth factor (Proia et al., 2008) that could be shared with glioma cells in which exert a proliferative action (Haley and Kim, 2014; Bian et al., 2000; Jimenez-Pascual et al., 2020).

### 4.3 Crosstalk *via* gap junctions

Astrocytes are highly interconnected through gap junctions that allow for fast ions and metabolites exchange. Gap junctions are made up of two hemichannels, each expressed on a different cell. Each hemichannel, called connexin, consists of six protein subunits (Scott et al., 2012). Connexin 43 (Cx43) represents the most abundant subunit on astrocytes (Rash et al., 2001, Xing et al., 2019). Even if Cx43 is overexpressed in the tumoral core, contributing to the increase of GBM-GBM cell communication, it is also over expressed in a subset of reactive astrocytes close to tumor cells. Selective deletion of Cx43 in reactive astrocytes attenuates glioma invasion *in vivo* (Sin et al., 2016). In line with this finding, it has been found that glioma-astrocyte gap junctions enable the transfer from GBM cells to astrocytes of many miRs (such as miR19) that downregulates the expression of cadherins, integrins, focal adhesion kinases, and other adhesion molecules. This process favors a reduced adhesion of astrocytes to the basement membrane, thereby opening a gateway that favors tumor cell invasion (McCutcheon and Spray, 2022). In addition, Cx43 mediates the transfer of cGAMP from brain metastatic cancer cells to astrocytes inducing the release of factors (such as TNFα) that activate the NF-kB pathways on cancer cells (Chen Q. et al., 2016), thus promoting metastasis progression (Wang et al., 2017). Cx43 gap junctions between metastatic brain cancer cells and astrocytes are favored by the over expression of the brain specific cell adhesion molecule protocadherin 7 (PCDH7) in metastatic cells (Chen Q. et al., 2016).

The expression of genes related to drug resistance, anti-apoptosis and survival in glioma cells also depends on genetic material that is transferred from astrocytes to glioma cells through gap junctions (Lin et al., 2016). Among these genetic materials are also microRNAs such as miR5096 that can activate pro-invasive pathways in cancer cells (Hong et al., 2015), or miR152-3p that can reduce cell migration and invasion of glioma cells (Fukuda et al., 2021).

The tight junctions between astrocytes and vessel smooth muscle cells are unsettled by tumor cells that creep between the two healthy cell types (Watkins et al., 2014). In this manner, tumor cells take control of vessel tone by modulating K^+^ efflux, and thus dilate or constrict arterioles by the same mechanism used by astrocytes (Zonta et al., 2003).

### 4.4 Crosstalk *via* tunneling nanotubes

Another direct contact between astrocytes and glioma cells is represented by tunneling nanotubes (TNTs), thin and long protuberances (up to 550 μm) of the cell cytoplasm. TNTs allow the transfer of ions, molecules, and organelles from the donor to the target cell (Davis and Sowinski, 2008). Astrocyte-glioma nanotubes initiating from astrocytes are able to reduce the proliferation of tumor cells (Zhang and Zhang, 2015). Tumoral TNTs, called tumor microtubes, are structurally different showing less F-actin content, and being long-live and thicker than non-tumoral TNTs. They contribute to tumor growth, by distributing potentially toxic material for tumor cells, such as calcium (Li et al., 2020), to neighboring cells keeping its intracellular levels within non-lethal limits (Osswald et al., 2015). Besides TNTs can translocate larger structures such as cellular organelles that can change the functionality of recipient cells. For example, tumor microtubes transfer mitochondria from glioma to healthy astrocytes. These mitochondria transform the metabolism of recipient cells (i.e., non-neoplastic astrocytes) into a tumor-like metabolism, especially with regard to the utilization of glutamine as major energy source instead of glucose and lipids used by healthy astrocytes (Valdebenito et al., 2021); thus, non-neoplastic astrocytes become resistant to the hypoxic environment induced by the fast proliferation of tumor cells (Beppu et al., 2002). The *in vivo* discovery of a microtubes-mediated functional coupling between GBM cells and astrocytes is recent (Venkataramani et al., 2022) and highlights a potentially relevant aspect for diagnostic and therapeutic purposes.

## 5 Non-neoplastic astrocytes counteract GBM

Although non-neoplastic astrocytes engage direct and indirect dialogue with glioma cells contributing to tumor progression, recent data suggest that these cells can also represent key elements to contrast tumor progression (Fletcher-Sananikone et al., 2021; Serpe et al., 2022).

One strategy to counteract glioma might be represented by EVs released by non-neoplastic astrocytes. Recently (Serpe et al., 2022) we have found that EVs derived from glioma-stimulated astrocytes increase glioma proliferation and *in vivo* tumor volume. In contrast, EVs derived from normal astrocytes are able, *in vitro*, to reduce glioma cell proliferation, migration and invasion capability. Moreover *in vivo*, administration of these EVs reduces glioma tumor volume, proliferation rate, and in addition, impairs cell volume regulation. We found that among molecules transported by these EVs there is miR124. Such molecules are able to reduce the expression in glioma cells of LRCC8C protein, a subunit of the volume regulated anion channels (VRACs) that play a role in the modification of cell volume, necessary for cell migration and proliferation. Glioma cells kill surrounding neurons by releasing glutamate in order to create the necessary space to grow (Ye and Sontheimer, 1999). VRACs are also permeable to excitatory amino acids including glutamate (Feustel et al., 2004); thus, we speculate that the reduction of VRAC expression might contribute to reduction in glioma release of glutamate.

Another strategy could be the use of non-neoplastic astrocytes as therapeutic targets. After surgical resection of glioma, the standard of care is patients’ treatment with up to 60 Gy of fractionated ionized irradiation (IR) with concurrent adjuvant chemotherapy, such as Temozolomide (Stupp et al., 2005). However, despite the positive response following IR therapy, later on the recurrence of a more invasive and resistant glioma lead to a fatal prognosis (Osuka and Van Meir, 2017, Scoccianti et al., 2021). Recently, it has been demonstrated *in vivo*, using syngeneic GL261 mouse model of glioma, and *in vitro*, using coculture of normal astrocytes and GL261 glioma cells, that upon irradiation normal astrocytes became senescent and release factors, including HGF. HGF then activates the Met receptor on glioma, promoting tumor invasiveness. *In vivo*, blocking Met activation by pharmacological approach results in attenuation of tumor growth and increased mice survival. Further, the elimination of senescent astrocytes using a senolytic drug results in delayed tumor growth in pre-irradiated brains (Fletcher-Sananikone et al., 2021).

## 6 Conclusion

In this Mini Review different strategies of the crosstalk between surrounding non-neoplastic astrocytes and brain primary or metastatic tumor cells have been reported. Astrocytes react to brain tumor cells engaging in undirect and direct dialogues mainly to support the tumor growth. Astrocytic release of soluble factors fosters migration, invasion, and growth of primary brain tumor cells. In brain metastasis these factors, released by astrocytes close to the extravasated cancer cells, support the brain entering.

Extracellular vesicles released by primary tumor cells promote astrocytic polarization toward an immunosuppressive phenotype and astrocytic release of soluble factors that contribute to the tumor progression. EVs released by astrocytes transfer miRs and growth factors into metastatic cancer cells that support metastatization into the brain.

Direct exchange of molecules occurs through gap junctions or tunneling nanotubes between astrocytes and brain tumor cells also support their crosstalk.

However, recent data suggest that EVs from non-neoplastic astrocytes or selective elimination of non-neoplastic astrocytes may be used to counteract brain tumors. Considering these findings, it is possible to speculate that in the future, administration of EVs obtained from non-neoplastic astrocytes (for example derived from patient differentiated IPSCs), and/or targeting non-neoplastic astrocytes by using senolytic therapy, could represent an alternative or coadiuvant therapeutic approach to limit brain tumor progression and to contrast glioma recurrence.

## Author contributions

MC: Conceptualization, Data curation, Supervision, Writing – original draft, Writing – review & editing, Funding acquisition. CL: Funding acquisition, Supervision, Writing – review & editing. FT: Conceptualization, Data curation, Supervision, Writing – original draft, Writing – review & editing.
